# Improving spatial nitrogen dioxide prediction using diffusion tubes: A case study in West Central Scotland

**DOI:** 10.1016/j.atmosenv.2015.08.009

**Published:** 2015-10

**Authors:** Francesca Pannullo, Duncan Lee, Eugene Waclawski, Alastair H. Leyland

**Affiliations:** a MRC∣CSO Social and Public Health Science Unit, University of Glasgow, 200 Renfield Street, Glasgow, G2 3QB, UK; b School of Mathematics and Statistics, University of Glasgow, Glasgow, G12 8QW, UK; c Public Health, University of Glasgow, G12 8SJ, UK

**Keywords:** Bayesian fusion modelling and prediction, Nitrogen dioxide, Spatial prediction

## Abstract

It has been well documented that air pollution adversely affects health, and epidemiological pollution-health studies utilise pollution data from automatic monitors. However, these automatic monitors are small in number and hence spatially sparse, which does not allow an accurate representation of the spatial variation in pollution concentrations required for these epidemiological health studies. Nitrogen dioxide (NO_2_) diffusion tubes are also used to measure concentrations, and due to their lower cost compared to automatic monitors are much more prevalent. However, even combining both data sets still does not provide sufficient spatial coverage of NO_2_ for epidemiological studies, and modelled concentrations on a regular grid from atmospheric dispersion models are also available. This paper proposes the first modelling approach to using all three sources of NO_2_ data to make fine scale spatial predictions for use in epidemiological health studies. We propose a geostatistical fusion model that regresses combined NO_2_ concentrations from both automatic monitors and diffusion tubes against modelled NO_2_ concentrations from an atmospheric dispersion model in order to predict fine scale NO_2_ concentrations across our West Central Scotland study region. Our model exhibits a 47% improvement in fine scale spatial prediction of NO_2_ compared to using the automatic monitors alone, and we use it to predict NO_2_ concentrations across West Central Scotland in 2006.

## Highlights

• Monitors and diffusion tubes that measure NO_2_ are spatially sparse.
• Modelled concentrations are also available, but are known to contain biases.
• We utilise all three sources of data to predict fine scale NO_2_ concentrations.
• A geostatistical fusion model is utilised implemented within a Bayesian setting.
• Addition of diffusion tubes enhances the predictive performance of fine scale NO_2_.

## 1 Introduction

The relationship between air pollution concentrations and ill health has been well documented in the past two decades, with epidemiological studies focussing on the effects of both short-term and long-term exposure. The most common study design is an ecological time series study, such as Omori et al. (2003) and Moolgavkar et al. (2013), which examines the effects of short-term exposure by regressing routinely available air pollution and disease data collected at daily intervals. The health impact of long-term exposure has typically been estimated using cohort studies such as Dockery et al. (1993) and Jerrett et al. (2009), but they are expensive and time consuming to implement due to the length of time required for monitoring the health status of the cohort. Therefore, spatial ecological studies such as Lee et al. (2009) and Haining et al. (2010) have recently been used to estimate the long-term effects of air pollution on human health, which regress variation in disease risk and air pollution across small areal units such as electoral wards using routinely collected health data.

The health data considered in these studies have included mortality (Kinney and Ozkaynak, 1991) and morbidity, such as hospital admissions (Willocks et al., 2012), for a number of common diseases, such as respiratory (Atkinson et al., 2001) and cardiovascular conditions (Larrieu et al., 2007). Numerous pollutants have been associated with disease in these studies, including carbon monoxide (CO, (Villeneuve et al., 2003)), nitrogen dioxide (NO_2_, (Raaschou-Nielsen et al., 2012)), ozone (O_3_, (Tao et al., 2012)), particulate matter (such as PM_10_ (Rushworth et al., 2014) and PM_2.5_, (Cesaroni et al., 2013)) and sulphur dioxide (SO_2_, (Wong et al., 2008)). The pollution data used in these studies typically come from a small number of automatic monitors, each of which measures concentrations of the above pollutants at a single point in space. However, the number of monitors is few and their geographical positioning is sparse, which does not allow an accurate representation of the spatial variation in pollution concentrations required for the epidemiological studies, particularly cohort and spatial ecological studies. For cohort studies, concentrations are required at the residence of each member in the cohort, while for spatial ecological studies concentrations are required for each spatial unit at which health data are available. These fine scale pollution data are not available, for example in our Glasgow region there are only 16 monitors covering the 368 square kilometre study region. Therefore, inexpensive non-automatic diffusion tubes are also used to measure concentrations of NO_2_, and due to their lower cost compared with automatic monitors they are more prevalent. For example, in the Glasgow study region considered here there are 230 diffusion tubes, which thus provides greatly enhanced spatial coverage compared with using the 16 automatic monitors alone.

**Fig. 1:**
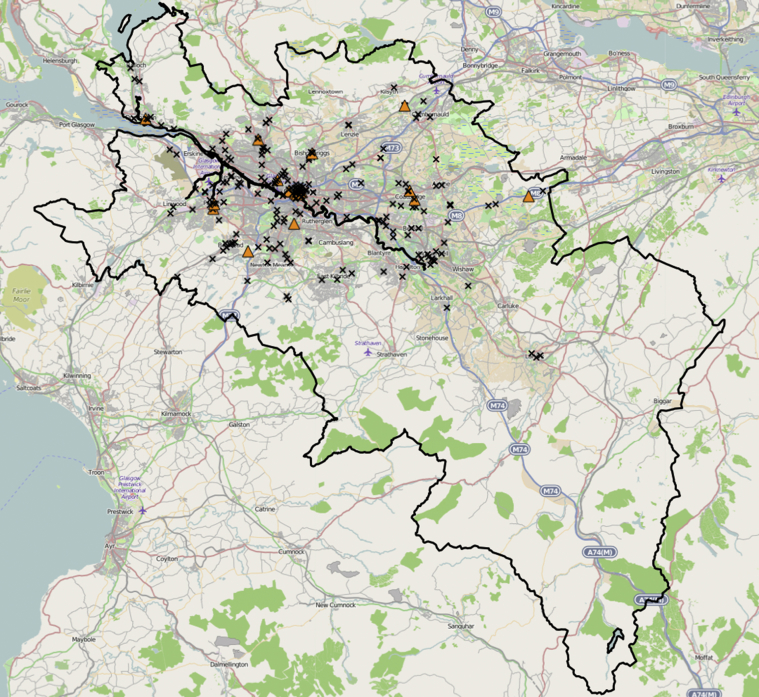
This map showcases the locations of the measured (automatic monitor and diffusion tube) NO_2_ data for 2006 with the outline of our West Central Scotland study region. Crosses denote diffusion tubes, and triangles denote automatic monitors.

However, combining these two data sets still does not give complete spatial coverage of a study region, which is illustrated in our case study in Fig. 1. Therefore, modelled concentrations from atmospheric dispersion models are increasingly being used in health studies (Naess et al., 2007), as they provide estimated concentrations on a regular grid and thus have complete spatial coverage of the study region. However, these modelled concentrations are known to contain biases (Berrocal et al., 2010a), and are not as accurate as the measured pollution data. Therefore, there has been recent research interest in statistical fusion models (Berrocal et al., 2010b; Fuentes and Raftery, 2005), which use both the measured and modelled concentrations to predict pollution at fine spatial and temporal scales. There are two main types of statistical fusion models, namely: the regression approach, and the latent process approach. The regression approach, as utilised by Berrocal et al. (2010a, 2010b), Bruno et al. (2013), combines monitored and modelled concentrations by regressing the concentrations from the monitoring stations against the modelled concentrations via a spatially varying linear regression. The latent process approach, utilised by Fuentes and Raftery (2005), Fuentes et al. (2008), McMillan et al. (2009), Sahu et al. (2010), represents the true environmental factor, and drives both the observed and modelled data assuming that both the modelled and observed data provide good information about the same underlying process. These fusion models correct for the biases inherent in atmospheric dispersion models, and also provide associated measures of uncertainty for the predictions, which are typically not available from dispersion models. However, the majority of these models only make use of measured data from automatic monitors, which as previously discussed are spatially sparse.

Therefore in this paper we propose a geostatistical fusion model, that regresses the combined NO_2_ concentrations from both automatic monitors and diffusion tubes against modelled NO_2_ pollution data from an atmospheric dispersion model. This model is implemented within a Bayesian setting and predicts NO_2_ concentrations across the Glasgow region, for use in a future health study. This is thus the first paper to demonstrate the dramatic improvement in fine scale spatial prediction of NO_2_ that can be obtained by using abundant diffusion tube data that is relatively inexpensive to collect in addition to the small numbers of automatic monitors.

The remainder of this paper is organised as follows. Section 2 describes the study design of our Glasgow case study, specifically the spatial extent of the region of interest and the NO_2_ and covariate data. Section 3 presents the geostatistical fusion model for predicting NO_2_ concentrations across the study region proposed here, and discusses its implementation. Section 4 presents the results of our analyses, including a model validation exercise that compares our proposed model against a number of other candidate models, and a fine scale prediction of NO_2_ across the Glasgow region. Finally, Section 5 provides a concluding discussion.

## 2 Study design

### 2.1 Study region

Our study region is centred around the Greater Glasgow conurbation, which is the largest city in Scotland, UK. The Glasgow conurbation contains just under one quarter of the total Scottish population, equating to around 1.1 million people, with a land area of around 368 km^2^. Seven local authorities comprise the study region, namely: East Renfrewshire, Glasgow City, North Lanarkshire, Renfrewshire, South Lanarkshire, and West Dunbartonshire. These local authorities have been selected because they cover the city of Glasgow and the wider area, collectively known as *West Central Scotland*, and include both urban and rural areas leading to a wide variation in pollution concentrations across the study region.

### 2.2 Air pollution data

The air pollution data comprise annual mean concentrations of nitrogen dioxide (NO_2_, measured in *μ*gm^−3^) in 2006, for which two sources of data are available. The first source is measured data at fixed points in space, which come from both automatic monitoring stations and non-automatic diffusion tubes. NO_2_ concentrations from the automatic monitors were downloaded from the Scottish Air Quality website (www.scottishairquality.co.uk), while the non-automatic diffusion tube data were obtained on request from each local authority. The accuracy of the diffusion tubes vary depending on numerous factors, such as the preparation methodology used, and are therefore calibrated using a bias-adjustment factor obtained from co-location studies. These concentrations are measured at 246 sites across the study region, of which 230 are diffusion tubes and 16 are automatic monitors. The locations of these sites within the study region are displayed in Fig. 1, where the diffusion tubes are displayed as crosses and the automatic monitors are presented as triangles. Summary statistics for the measured data are shown in Table 1. These statistics highlight that the distribution of concentrations across the study region are slightly higher for monitors compared to diffusion tubes with median values of 34.55 μgm^−3^ and 29.95 μgm^−3^ respectively. This could be due to the local authorities placing automatic monitors where they have a compliance problem with EU pollution standards.

Fig. 1 shows that the measured data are sparse, and do not provide complete spatial coverage of the study region. In addition, the automatic monitors and diffusion tubes are also likely to be preferentially located in areas where pollution is thought to exceed EU standards, therefore producing an inflated picture of area wide pollution concentrations. Furthermore, the monitoring network often suffers from missing data, arising from monitors and tubes becoming faulty. Nevertheless, as the concentrations are directly measured they provide close to the true value with little measurement error.

**Fig. 2:**
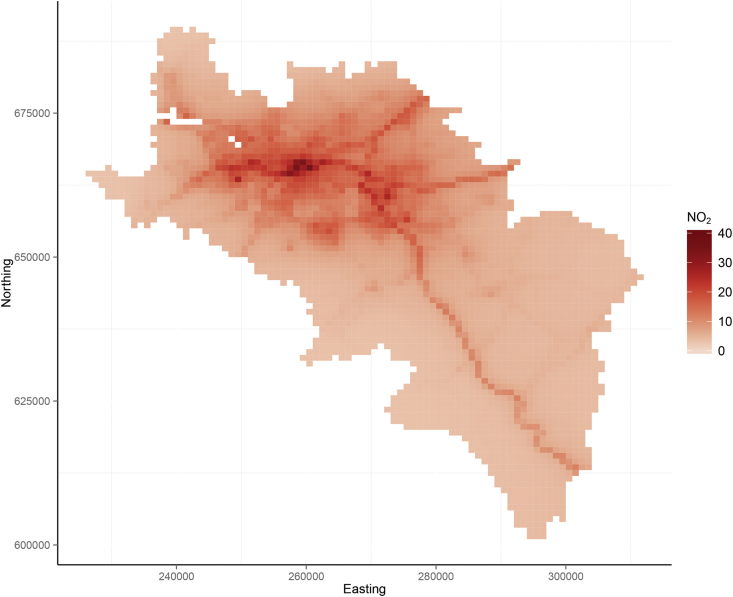
This map showcases the 2006 modelled-PCM NO_2_ (μgm^−3^) concentrations from an atmospheric dispersion model across West Central Scotland at a 1 km resolution.

The second source of data are modelled concentrations based on the UK Pollution Climate Mapping (PCM) approach, provided by the Department for Environment, Food and Rural Affairs (DEFRA) (http://uk-air.defra.gov.uk/). These data are modelled-PCM yearly mean background concentrations in *μ*gm^−3^ at a 1 km × 1 km resolution, which hence provide complete spatial coverage across the study area with no missing data. However, modelled-PCM concentrations such as these are known to contain biases due to being uncalibrated, and no measure of variability is available to quantify the level of uncertainty in these estimates. These data are displayed in Fig. 2, where the city of Glasgow and the main motorway network are easy to see. These annual mean concentrations range between 3.021 μgm^−3^ and 34.760 μgm^−3^ with a mean value of 7.632 μgm^−3^ across the study region. These concentrations are lower compared to the measured data as they are average background concentrations over a 1 km grid, rather than relating to specific pollution sources such as roads.

### 2.3 Covariate data

A number of covariates were considered in this study. Firstly, an indicator variable was included in order to distinguish the pollution concentrations measured from the two types of equipment: automatic monitors and diffusion tubes. Secondly, the local environment in which each of the automatic monitors and diffusion tubes were located was also recorded. These local environments include kerbside (located within 1 m of the kerb on a busy road), roadside (located between 1 m and 5 m of a busy road), urban background (located away from direct sources usually in urban residential areas), rural (countryside location far from roads, populated and industrial areas) and ‘special’ (located at Glasgow airport and at industrial sources). In total, there are 142 roadside, 34 kerbside, 8 special, 60 urban background, and 2 rural sites. Thirdly, to enable spatial prediction an urban–rural variable was constructed, which classifies each prediction location according to the Scottish Government 6 fold Urban Rural Classification (http://www.scotland.gov.uk/). Each location was considered urban if it was situated in a built-up area containing more than 10,000 people, and rural otherwise.

## 3 Statistical methods

This section presents the statistical fusion model proposed in this paper for predicting NO_2_ concentrations across the West of Scotland region, using both the measured (automatic monitors and diffusion tubes) and modelled-PCM pollution data. The first section presents the statistical fusion model, while the second outlines the prediction methodology. The model is fitted in a Bayesian setting, with inference based on Markov chain Monte Carlo (McMC) simulation.

### 3.1 Spatial fusion model

Let **Y** = (*Y*(**s**_1_),…,*Y*(**s**_*m*_)) denote the vector of (natural) log-transformed NO_2_ concentrations from both the automatic monitors and diffusion tubes at spatial locations (**s**_1_,…,**s**_*m*_), where the latter are measured as Eastings and Northings in metres. The NO_2_ data are log-transformed because they are non-negative and skewed to the right, and exploratory analyses suggested that a log-transformation improved the fit of the resulting regression models. These measured NO_2_ concentrations are regressed against a matrix of *p* covariates denoted by **Z** = (**z**(**s**_1_)^*T*^,…, **z**(**s**_*m*_)^*T*^)^*T*^, where the values relating to spatial location **s**_*i*_ are denoted by **z**(**s**_*i*_)^*T*^ = (1, *z*_2_(**s**_*i*_),…, *z*_*p*_(**s**_*i*_)). This covariate matrix includes a column of ones for the intercept term, the (natural) log-transformed modelled-PCM concentrations and any other relevant covariates, such as the local environment in which the observation is located (e.g. roadside, urban background, etc). Thus, this model fuses the monitored and modelled-PCM pollution data via a linear regression relationship.

We propose a Bayesian geostatistical fusion model for these data, which relates the modelled-PCM and measured NO_2_ concentrations using the following equation:

(1) $$Y\left(s_{i}\right)∼\text{N}\left(z{\left(s_{i}\right)}^{T}\alpha +\phi \left(s_{i}\right),\nu^{2}\sigma^{2}\right),\;i=1,\ldots ,m\text{.}$$

The mean function is a linear combination of a covariate component **z**(**s**_*i*_)^*T*^***α***, with associated regression parameters ***α*** = (*α*_1_,…,*α*_*p*_), and a spatial random effect *ϕ*(**s**_*i*_). The regression parameters ***α*** are assigned a weakly informative multivariate Gaussian prior with a mean of zero and a large diagonal variance matrix. The spatial random effects for all *m* locations are collectively denoted by ***ϕ*** = (*ϕ*(**s**_1_),…,*ϕ*(**s**_*m*_)), and allow for any unmeasured spatial autocorrelation in the measured NO_2_ data after the covariate effects have been accounted for. Here we model their spatial autocorrelation using the formulation

(2) $$\begin{array}{l} \phi ∼\text{N}\left(0,\sigma^{2}\text{Σ}\left(\rho \right)\right),\\ \sigma^{2}∼\text{Inverse}-\text{Gamma}\left(a,b\right),\\ \rho ∼\text{Discrete}\;\text{Uniform}\left(\rho_{1},\ldots ,\rho_{r}\right)\text{.} \end{array}$$

The random effects are assumed to come from a multivariate Gaussian distribution with mean zero, variance *σ*^2^, and a spatial correlation matrix Σ(*ρ*). This matrix is defined by an isotropic exponential correlation function of the distance between any two locations, that is Σ(*ρ*) = exp(−*ρD*). Here *D* is an *m* × *m* distance matrix, where the *ij* th element $D_{ij}=\left\|s_{i}-s_{j}\right\|$ is the Euclidean Distance between spatial location (**s**_*i*_,**s**_*j*_). The exponential model was chosen for simplicity and because it is the most commonly used model in the geostatistical literature (see for example (Vicedo-Cabrera et al., 2013)). A conjugate inverse-gamma prior was specified for the spatial variance *σ*^2^, where (*a* = *b* = 0.001) were chosen to be non-informative. Here *ρ* is the spatial decay parameter, which controls the rate at which the spatial autocorrelation between a pair of sites declines as the distance between them increases. We specify a discrete uniform prior with a large range for *ρ* as suggested by Diggle and Ribeiro (2007) for computational efficiency, because this ensures that the correlation matrix Σ(*ρ*) only needs to be inverted *r* = 50 times, once for each of the candidate values *ρ*_1_,…,*ρ*_*r*_, rather than at every step of the McMC algorithm. Finally, the nugget effect, that is the amount of non-spatial variation or measurement error, is controlled by *ν*^2^*σ*^2^, the product of the spatial variance parameter and the noise-to-signal ratio *ν*^2^. This latter parameter is assigned a uniform prior on the unit interval, as the nugget effect is expected to be smaller than the amount of spatial variation for these data.

### 3.2 Spatial prediction

Bayesian spatial prediction using kriging is a natural extension in the Bayesian paradigm to estimating the parameters **Θ** = (***α***, ***ϕ***, *ν*^2^, *σ*^2^), and is implemented as a two-step procedure within the McMC algorithm. In model (1) and (2), spatial autocorrelation is induced into the mean function through the random effects ***ϕ***. Therefore, the first step in spatial prediction generates the random effects at *N* prediction locations $s^{*}=\left(s_{1}^{*},\ldots ,s_{N}^{*}\right)$ using multivariate Gaussian theory. Specifically, the random effects at the prediction locations $\phi^{*}=\left(\phi \left(s_{1}^{*}\right),\ldots ,\phi \left(s_{N}^{*}\right)\right)$ are sampled from their conditional distribution given ***ϕ***, that is

(3) $$\phi^{*}|\phi ∼\text{N}\left(E\left[\phi^{*}|\phi \right],\mathrm{Var}\;\left[\phi^{*}|\phi \right]\right)\text{.}$$

The mean and variance are given by

$$E\left[\phi^{*}|\phi \right]=C_{Z}{\left(s^{*},\rho \right)}^{T}\Sigma^{*}{\left(\rho \right)}^{-1}\phi$$

and

$$\mathrm{Var}\left[\phi^{*}|\phi \right]=\sigma^{2}\left[\Sigma^{*}\left(\rho \right)-C_{Z}{\left(s^{*},\rho \right)}^{T}\Sigma^{*}{\left(\rho \right)}^{-1}C_{Z}\left(s^{*},\rho \right)\right]\text{,}$$

where Σ^∗^(*ρ*) is an *N* × *N* spatial correlation matrix for the *N* prediction locations and *C*_*Z*_(**s**^∗^,*ρ*) is an *N* × *m* spatial correlation matrix between the prediction and the observation locations. These equations are equivalent to ordinary kriging.

The second step generates the predicted value of $Y\left(s_{i}^{*}\right)$ for the *N* prediction locations as

(4) $$Y\left(s_{i}^{*}\right)∼\text{N}\left(z{\left(s_{i}^{*}\right)}^{T}\alpha +\phi \left(s_{i}^{*}\right),\sigma^{2}\nu^{2}\right)\text{,}$$

where $z\left(s_{i}^{*}\right)$ denotes the matrix of covariates at the *N* prediction locations.

Leave-one-out cross-validation is performed in order to assess the quality of the predictions, which removes each measured data point in turn and then predicts its value from the remainder of the data. The accuracy of the predictions compared to the measured NO_2_ concentrations are compared using three statistics, namely: bias, root mean square prediction error (RMSPE) and the coverage probabilities of the 95% prediction intervals. The bias is given by

(5) $$\text{Bias}=\frac{1}{m}\sum_{i=1}^{m}\left(Y\left(s_{i}^{*}\right)-Y\left(s_{i}\right)\right)\text{,}$$

where a bias of zero means the predictions are the correct size on average. The RMSPE is given by

(6) $$\text{RMSPE}=\sqrt{\frac{1}{m}\sum_{i=1}^{m}{\left(Y\left(s_{i}^{*}\right)-Y\left(s_{i}\right)\right)}^{2}}\text{,}$$

and for unbiased predictions it measures the amount of variation in the predictions around the true value, with smaller values indicating more precise estimation. Finally, a 95% prediction interval is computed for each predicted NO_2_ concentration, and the coverage probability of a model is the percentage of these prediction intervals that contain the true value. The prediction intervals are the correct width if 95% of these intervals contain the true value.

### 3.3 Inference

The McMC simulation algorithm produces a set of *M* samples for each of the model parameters **Θ** = (***α***, ***ϕ***, *ν*^2^, *σ*^2^, *ρ*), based on a mixture of Gibbs sampling and Metropolis–Hastings steps. The results from our study are based on 10,000 posterior samples generated from one Markov chain, which has been burnt-in until convergence by assessing the stability of trace plots of the McMC samples from selected parameters. The McMC simulation algorithm was implemented within the R (R Development Core Team, 2014) statistical programming language using functions written by the authors.

## 4 Results

This section presents the results of applying the statistical fusion model proposed in Section 3 to the Glasgow case study outlined in Section 2. Section 4.1 presents a validation study, which compares the appropriateness of our proposed model against a number of alternative models in terms of both model structure and covariate choice. Section 4.2 demonstrates the advantages of using diffusion tube data for fine scale spatial prediction, by comparing predictive accuracy against using the automatic monitors alone. Finally, Section 4.3 uses the best performing model from the previous two sections to predict yearly average NO_2_ concentrations at a 1 km grid square resolution across the study region, with associated standard errors.

### 4.1 Validation study 1: model structure and covariate choice

In this validation study we compare the predictive performance of a number of different model specifications, focusing on the utility of allowing for spatial autocorrelation in the data, the approach to parameter estimation adopted for the model, and the choice of covariates.

The results of our validation study are presented in Table 2, for nine different models. The top panel of the table compares the utility of allowing for spatial autocorrelation in the data and the estimation approach taken, while the bottom panel shows a sensitivity analysis to the choice of covariates. In all cases the models are unbiased, as the biases are all close to zero ranging between −0.0001 and 0.356. Model 1 is the Bayesian model described in Section 3, which includes the log-transformed modelled-PCM NO_2_ concentrations (*log modelled*), an indicator for the type of observed data (automatic monitor or non-automatic diffusion tube, *monitor/tube*), and the local environment in which each observation resides (e.g. roadside, urban background, etc, *environment*) as covariates. Model 2 is the same as Model 1 except that inference is performed using restricted maximum likelihood estimation, and the RMSPE values are almost identical. The differences are in the coverage probabilities, with the Bayesian estimation having wider and more appropriate prediction intervals (coverages differ by around 1%) than under likelihood based estimation. This small difference is because when using restricted maximum likelihood the estimated model parameters are assumed to be fixed and known when making the predictions, thus underestimating the amount of uncertainty in the data. In contrast, the Bayesian model allows for uncertainty in the estimated model parameters when making predictions, thus explaining its wider prediction intervals. Model 3 also uses maximum likelihood estimation, but naively ignores the spatial autocorrelation present in the data. This model shows around a 5% increase in RMSPE compared with Model 1, suggesting that ignoring the spatial autocorrelation in the data results in poorer predictive performance.

The bottom panel of Table 2 shows a comparison of different combinations of covariates, which are summarised below.

**Model 1 –***log modelled + monitor/tube + environment*
**Model 4 –***log modelled*
**Model 5 –***log modelled* + *monitor/tube*
**Model 6** – *log modelled + environment*
**Model 7** – *monitor/tube + environment*
**Model 8** – *log modelled + monitor/tube + environment + log modelled:easting + log modelled:northing*
**Model 9** – *log modelled + environment + log modelled:easting + log modelled:northing*

In all cases the Bayesian fusion model described in Section 3 is used. These results show two main points. Firstly, the *log modelled* and *environment* variables are important for accurate NO_2_ prediction, which is evidenced by an increase in RMSPE for Models 4, 5 and 7 compared with Model 1. The bias and RMSPE are greatest for Model 4, because it does not include any covariates to distinguish between measurements made at the roadside, kerbside, rural or background environments. The *log modelled* variable is a spatially smooth covariate with no adjustment for the environment, so it cannot capture higher measurements at the roadside and lower background measurements. It therefore tends to overestimate the concentrations, which is evidenced by its higher bias and RMSPE. The importance of the *log modelled* covariate is clear given by the improved RMSPE in Model 1 (0.257) compared to Model 7 (0.276), while the *environment* variable is important because it distinguishes between observations at roadside and background environments, which will have a large impact on the measured NO_2_ value which is largely driven by traffic sources. Secondly, including the *monitor/tube* variable does not lead to improved NO_2_ prediction, as the RMSPE of Model 6 is 0.258 compared to 0.257 for Model 1. This can also be shown in Table 3, which displays the posterior medians and 95% credible intervals for the main parameters in Model 1. NO_2_ concentrations recorded by automatic monitors are slightly higher compared to NO_2_ concentrations recorded by diffusion tubes as the posterior median is positive, however the relationship is very weak as the 95% CI's lower bound is close to zero. For the environment variable, rural, special and urban background sites have substantially lower NO_2_ concentrations compared to kerbside sites; however even though roadside sites have lower NO_2_ pollution levels compared to kerbside sites, the relationship is quite weak as the upper bound for the credible interval is just below one, which is not surprising since roadside and kerbside sites both measure pollution at the roadside.

The bias and RMSPE are also computed for the modelled-PCM concentrations, thus allowing the improvement in predictive performance from our models to be observed. Since the modelled-PCM concentrations are background concentrations, we include an adjustment for sites measured at roadside and kerbside environments. The results show that even adjusting the modelled-PCM concentrations for roadside and kerbside environments (*roadside/otherwise*), the modelled-PCM concentrations are not as good for predicting NO_2_ concentrations with a RMSPE of 0.337 (and a bias of −0.068) compared to a RMSPE of 0.258 for Model 6.

Each of the models described above assume the effect of the modelled-PCM concentrations is constant across space. This necessarily might not be the case as the effect may vary depending on the spatial location. Therefore, to allow flexibility in the effect of the modelled-PCM concentrations to vary across space, Models 8 and 9 contain an interaction term between the *log modelled* variable and the easting and northing coordinates of the location of the automatic monitors and diffusion tubes, given as *log modelled:easting* and *log modelled:northing*. Model 9 is the same as Model 8 except it does not contain the *monitor/tube* covariate. Both models are unbiased, and have the same RMSPE of 0.255 and coverage probability of 94.715%, indicating that again the *monitor/tube* variable does not lead to improved NO_2_ prediction. Even though the bias in Model 9 (0.013) is slightly higher compared to Model 8 (0.009), Model 9 is a better model compared to Model 6 as it treats the effect of the modelled-PCM concentrations constant across space as the RMSPE decreases by around 1% and the coverage probability is closer to the nominal value of 95%. Therefore, the final model we consider here is Model 9, as it is more flexible compared to Model 6, and has improved performance, mainly in terms of coverage probability. We also considered allowing the effect of the modelled-PCM concentrations to vary as a quadratic surface in location added into Model 9 as *log modelled:easting*^2^ + *log modelled:northing*^2^, but this did not improve the results.

### 4.2 Validation study 2: data source

This second validation study investigates the effectiveness of using the diffusion tube data in addition to the automatic monitoring data for fine scale spatial prediction. We use Model 9 throughout this section, as Section 4.1 showed it had the best overall performance. In common with Section 4.1 we use leave-one-out cross-validation to assess predictive accuracy, again using bias, RMSPE and coverage probabilities to quantify prediction performance. We fit Model 9 to three different subsets of the data: only the 16 automatic monitors, only the 230 diffusion tubes, and the combined 246 observations. In each case the model is used to predict each of the 246 observations in turn.

The results of our second validation study are displayed in Table 4 for the three different subsets of the observed pollution data. In common with the results from the first validation study in Section 4.1, all three models are unbiased, as the biases are all close to zero ranging from 0.009 to 0.266 However, the predictive performance from using only the automatic monitors is markedly poorer than using either just the diffusion tubes or all the observations, which is evidenced by both its RMSPE and coverage probability. The RMSPE from using the monitors only is 0.478, which is greater by 48% (RMSPE of 0.249) and 47% (RMSPE of 0.255) than the corresponding values from using the diffusion tubes only and the combined data set. Additionally, the coverage probability when using the automatic monitors alone is over 99.5%, which is larger than the nominal 95% levels. This high coverage probability suggests that the prediction intervals are too wide, which is most likely due to a lack of data provided by the automatic monitors, thus resulting in poorer parameter estimation and higher uncertainty.

In contrast, the coverage probabilities from using just the diffusion tubes and all the observed data are close to their nominal 95% levels, while the RMSPE for the tube only model is 0.249 and 0.255 for the combined model. These results suggest that using the monitors in addition to the tubes does not lead to better predictive performance compared to using the tubes alone, as the two sets of results are essentially the same after allowing for random error. The reason for this is that some of the automatic monitors are co-located with the diffusion tubes so when the automatic monitors are included with the diffusion tubes, there is not a large increase in the number of observed data points. These results therefore demonstrate the effectiveness of using the diffusion tube data for predicting the NO_2_ concentrations at a fine spatial scale.

### 4.3 NO_2_ prediction

The model chosen to predict NO_2_ concentrations at each 1 × 1 km grid box resolution was Model 9; the Bayesian fusion model including both automatic monitors and diffusion tubes, with the log-transformed modelled-PCM concentrations and the local environment in which each automatic monitor and diffusion tube resides as covariates, while allowing the effect of the modelled-PCM concentrations to vary across space captured by an interaction term between the modelled-PCM concentrations and the easting and northing coordinates of the monitors and tubes. For spatial prediction purposes, grid boxes were predicted as urban background or rural. The remainder of the local environment locations (kerbside, roadside, and special) were not considered here because the NO_2_ concentrations we predict are averages over a 1 km × 1 km grid box, therefore NO_2_ measurements produced from the roadside are averaged out across the grid box. In addition, for health effects estimation, roadside concentrations are not representative of exposure levels as people do not spend large proportions of their time next to a road. The urban–rural variable discussed in Section 2.3 is used instead to predict each grid box as urban background or rural.

**Fig. 3:**
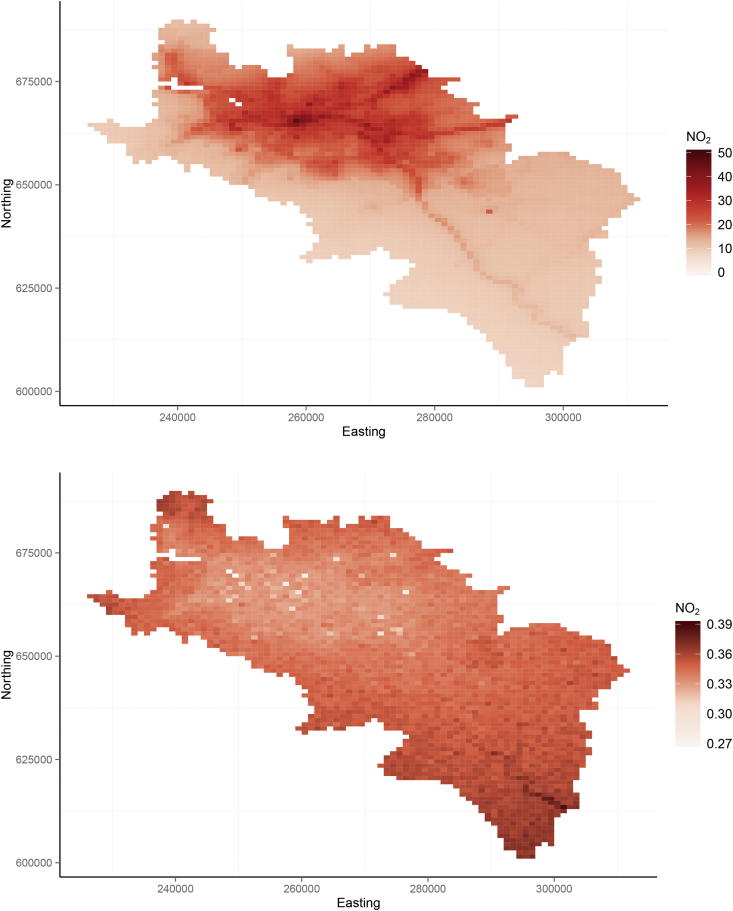
The top map shows the 2006 predicted NO_2_ (μgm^−3^) concentrations from Model 9 across West Central Scotland, while the bottom map shows the corresponding standard errors.

Fig. 3 displays the final predicted NO_2_ concentrations across the West of Scotland and their associated standard errors. The median of the 10,000 posterior samples was taken to be the Bayesian point estimate for these predicted NO_2_ concentrations. In common with the modelled-PCM concentrations in Fig. 2, the City of Glasgow and the main road network are visible and easy to see. Summary statistics for the predicted NO_2_ concentrations, their standard errors and the modelled-PCM concentrations are shown in Table 5 separately for urban and rural areas. These statistics highlight that the median concentration predicted from Model 9 is 23.000 μgm^−3^ for urban areas and 12.060 μgm^−3^ for rural areas, while for the modelled-PCM concentration the median value is 11.680 and 4.849 μgm^−3^ for urban and rural areas respectively. Therefore as we show in Table 2 that the predictions from Model 9 are unbiased, the modelled-PCM concentrations are likely to be underestimating NO_2_ concentrations across the West Central Scotland. However, the spatial pattern in the two sets of data are similar, with a correlation coefficient of 0.923 between the predictions from Model 9 and the modelled-PCM concentrations.

## 5 Discussion

This is the first paper to have demonstrated the improvement that can be made in the accuracy of fine scale spatial prediction of NO_2_ concentrations by using diffusion tube data in addition to the commonly used automatic monitors. Diffusion tubes are relatively inexpensive and thus more prevalent than automatic monitors in many urban environments, and the subsequent large increase in the number of spatial locations at which NO_2_ is measured leads to improvements in predictive performance. The Bayesian geostatistical fusion model we proposed links the observed and modelled-PCM NO_2_ concentrations via a regression relationship, and is similar to existing downscaling models used in the literature (Berrocal et al., 2010a, 2010b). The model performs fine scale spatial NO_2_ predictions that are unbiased and have appropriate width prediction intervals. Thus, this modelling framework should be useful for predicting NO_2_ concentrations in other urban environments.

The results from this paper have illustrated three key points. Firstly, using the diffusion tube data in addition to the automatic monitoring data enhances the predictive performance of fine scale NO_2_ concentrations, compared to using the automatic monitors alone. This is evidenced by a 47% reduction in RMSPE when utilising both sources of NO_2_ concentrations. This reduction in RMSPE is due to the increase in the number of observations used, resulting in more accurate parameter estimation and lower uncertainty. Furthermore, the bias reduced by a factor of 10 and the coverage improved by 5% when using both sets of measured pollution data. The latter is important because using the monitoring data alone resulted in too much predictive uncertainty. Secondly, using the modelled-PCM concentrations lead to improved spatial prediction, as a model containing the modelled-PCM concentrations surpassed the model without the modelled-PCM concentrations, with RMSPEs of 0.257 and 0.276 respectively, which is an increase of 7%. Finally, it is important to allow for spatial autocorrelation in the data, as the RMSPE increased by 5% compared to the model that did not take into account spatial autocorrelation. Furthermore, Bayesian methods allow for better uncertainty quantification than likelihood based estimation, as the coverage probability is closer to the nominal 95% level. The model chosen allowed the effect of the modelled-PCM concentrations to vary across space which showed a slight improvement over the model that assumed the effect was constant (RMSPE of 0.255 compared to 0.258). The main difference between these two models is in the coverage probability which was closer to the 95% nominal level. In absolute terms, our results do not show large differences between a model without using the diffusion tubes and a model with the diffusion tubes, but a 47% increase in performance highlights the utility of our approach.

The methodology proposed here has a number of limitations. The temporal resolution for our study was yearly, and it would be desirable to be able to apply the same methodology to higher resolution time periods such as daily. However, the diffusion tube data are only available as monthly averages, preventing the use of our approach at finer temporal scales. Furthermore, we predict background NO_2_ concentrations using the modelled-PCM NO_2_ data at a 1 km resolution, and thus our predictions are background concentrations that do not include local sources such as roads. In addition, we cannot predict NO_2_ concentrations at a finer spatial scale as the modelled-PCM concentrations are only available at a 1 km resolution.

There are numerous opportunities for future work from this paper. Our modelling approach can be linked with land-use regression in order to incorporate local traffic sources and predict NO_2_ at finer spatial scales. A natural extension to our modelling approach would be to include a temporal dimension for predicting NO_2_ concentrations across time, by utilising data for successive years. Furthermore, given NO_2_ is highly correlated with other pollutants such as PM_10_, the diffusion tube data could be used as a covariate for making spatial predictions for other related pollutants. Finally, our future research goal is to use the predicted NO_2_ concentrations from this paper in a spatial health impact study, to quantify effects that exposure to NO_2_ has on human health.

## Figures and Tables

**Table 1:** Summary statistics for the automatic monitoring and diffusion tube NO_2_ (μgm^−3^) data for 2006 across West Central Scotland.

	Monitors	Diffusion tubes
Min	10.00	9.00
25th Percentile	29.35	22.25
Median	34.55	29.95
Mean	38.31	31.63
75th Percentile	42.50	38.00
Max	89.00	86.10

**Table 2:** Bias (μgm^−3^), RMSPE (μgm^−3^) and coverage probability (%) results for the nine models compared in this section. The top panel displays the results for three models with different estimation methods, while the bottom panel displays the results for the six Bayesian models containing differing covariate combinations.

Model	Bias	RMSPE	Coverage
1	0.010	0.257	93.089
2	0.005	0.255	91.870
3	−0.0001	0.271	93.902

4	0.356	0.545	95.122
5	0.020	0.303	95.122
6	0.011	0.258	93.496
7	0.018	0.276	94.715
8	0.009	0.255	94.715
9	0.013	0.255	94.715

**Table 3:** Posterior medians and 95% credible intervals (CI) for selected parameters of Model 1, which is the full Bayesian model with log modelled, monitor/tube and environment as covariates. The diffusion tubes were taken as the reference category for monitor/tube and kerbside was taken as the reference category for environment. Results are also shown for the spatial variance *σ*^2^, noise-to-signal ratio *ν*^2^ and spatial decay parameter *ρ*.

Posterior median	Estimate	95% CI
Intercept	1.900	(1.564, 2.259)
Log modelled	0.594	(0.459, 0.708)
Monitor	0.125	(0.027, 0.238)
Roadside	−0.150	(−0.258, −0.042)
Rural	−1.021	(−1.585, −0.488)
Special	−0.390	(−0.630, −0.147)
Urban background	−0.531	(−0.659, −0.407)

*σ*^2^	0.057	(0.024, 0.077)
*ν*^2^	0.232	(0.064, 1.819)
*ρ*	12.852	(2.578, 53.946)

**Table 4:** Bias (μgm^−3^), RMSPE (μgm^−3^) and coverage probabilities (%) for the leave-one-out cross-validation of applying Model 9 to the three different sources of data.

Data source	Bias	RMSPE	Coverage
Monitors	0.266	0.478	99.594
Tubes	0.009	0.249	95.122
Monitors & Tubes	0.013	0.255	94.715

**Table 5:** Summary statistics for the 2006 modelled-PCM and predicted NO_2_ (μgm^−3^) concentrations from Model 9 with associated standard errors separately for urban and rural areas.

	Modelled-PCM NO_2_	Predicted NO_2_	Standard errors
Urban areas			
Min	3.207	112.570	0.273
25th Percentile	7.985	18.300	0.332
Median	11.680	22.650	0.336
Mean	12.040	23.000	0.336
75th Percentile	15.230	27.42	0.341
Max	34.760	46.400	0.364
Rural areas			
Min	3.021	8.028	0.321
25th Percentile	4.268	10.230	0.344
Median	4.849	12.060	0.349
Mean	5.575	13.020	0.349
75th Percentile	6.207	14.060	0.353
Max	18.090	32.090	0.387

## References

[bib1] Atkinson R.W., Anderson H.R., Sunyer J., Ayres J., Baccini M., Vonk J.M., Boumghar A., Forastiere F., Forsberg B., Touloumi G., Schwartz J., Katsouyanni K. (2001). Acute effects of particulate air pollution on respiratory admissions: results from APHEA 2 project. Air Pollution and Health: a European Approach. Am. J. Respir. Crit. Care Med..

[bib2] Berrocal V.J., Gelfand A.E., Holland D.M. (2010). A spatio-temporal downscaler for output from numerical models. J. Agric. Biol. Environ. Stat..

[bib3] Berrocal V.J., Gelfand A.E., Holland D.M. (2010). A bivariate space-time downscaler under space and time misalignment. Ann. Appl. Stat..

[bib4] Bruno F., Cocchi D., Greco F., Scardovi E. (2013). Spatial reconstruction of rainfall fields from rain gauge and radar data. Stoch. Environ. Res. Risk Assess..

[bib5] Cesaroni G., Badaloni C., Gariazzo C., Stafoggia M., Sozzi R., Davoli M., Forastiere F. (2013). Long-term exposure to urban air pollution and mortality in a cohort of more than a million adults in Rome. Environ. Health Perspect..

[bib6] Diggle P., Ribeiro P.J. (2007). Model-based Geostatistics.

[bib7] Dockery D.W., Pope C.A., Xu X., Spengler J.D., Ware J.H., Fay M.E., Ferris B.G., Speizer F.E. (1993). An association between air pollution and mortality in six US cities. N. Engl. J. Med..

[bib8] Fuentes M., Raftery A.E. (2005). Model evaluation and spatial interpolation by Bayesian combination of observations with outputs from numerical models. Biometrics.

[bib9] Fuentes M., Reich B., Lee G. (2008). Spatial–temporal mesoscale modeling of rainfall intensity using gage and radar data. Ann. Appl. Stat..

[bib10] Haining R., Li G., Maheswaran R., Blangiardo M., Law J., Best N., Richardson S. (2010). Inference from ecological models: estimating the relative risk of stroke from air pollution exposure using small area data. Spatial Spatio-temporal Epidemiol..

[bib11] Jerrett M., Finkelstein M.M., Brook J.R., Arain M.A., Kanaroglou P., Stieb D.M., Gilbert N.L., Verma D., Finkelstein N., Chapman K.R., Sears M.R. (2009). A cohort study of traffic-related air pollution and mortality in Toronto, Canada. Environ. Health Perspect..

[bib12] Kinney P.L., Ozkaynak H. (1991). Associations of daily mortality and air pollution in Los Angeles County. Environ. Res..

[bib13] Larrieu S., Jusot J.F., Blanchard M., Prouvost H., Declercq C., Fabre P., Pascal L., Tertre A.L., Wagner V., Rivière S., Chardon B., Borrelli D., Cassadou S., Eilstein D., Lefranc A. (2007). Short term effects of air pollution on hospitalizations for cardiovascular diseases in eight French cities: the PSAS program. Sci. Total Environ..

[bib14] Lee D., Ferguson C., Mitchell R. (2009). Air pollution and health in Scotland: a multicity study. Biostat. (Oxf. Engl.).

[bib15] McMillan N.J., Holland D.M., Morara M., Feng J. (2009). Combining numerical model output and particulate data using bayesian space-time modeling. Environmetrics.

[bib16] Moolgavkar S.H., Mcclellan R.O., Dewanji A., Turim J., Georg Luebeck E., Edwards M. (2013). Time-series analyses of air pollution and mortality in the united states: a subsampling approach. Environ. Health Perspect..

[bib17] Naess Ø., Piro F.N., Nafstad P., Smith G.D., Leyland A.H. (2007). Air pollution, social deprivation, and mortality: a multilevel cohort study. Epidemiology.

[bib18] Omori T., Fujimoto G., Yoshimura I., Nitta H., Ono M. (2003). Effects of particulate matter on daily mortality in 13 Japanese cities. J. Epidemiol./Jpn. Epidemiol. Assoc..

[bib19] Raaschou-Nielsen O., Andersen Z.J., Jensen S.S., Ketzel M., Sørensen M., Hansen J., Loft S., Tjønneland A., Overvad K. (2012). Traffic air pollution and mortality from cardiovascular disease and all causes: a Danish cohort study. Environ. Health.

[bib20] Rushworth A., Lee D., Mitchell R. (2014). A spatio-temporal model for estimating the long-term effects of air pollution on respiratory hospital admissions in Greater London. Spatial Spatio-temporal Epidemiol..

[bib21] R Development Core Team (2014). A Language and Environment for Statistical Computing.

[bib22] Sahu S.K., Gelfand A.E., Holland D.M. (2010). Fusing point and areal level space-time data with application to wet deposition. J. R. Stat. Soc. Ser. C (Appl. Stat.).

[bib23] Tao Y., Huang W., Huang X., Zhong L., Lu S.E., Li Y., Dai L., Zhang Y., Zhu T. (2012). Estimated acute effects of ambient ozone and nitrogen dioxide on mortality in the Pearl River Delta of southern China. Environ. Health Perspect..

[bib24] Vicedo-Cabrera A.M., Biggeri A., Grisotto L., Barbone F., Catelan D. (2013). A Bayesian kriging model for estimating residential exposure to air pollution of children living in a high-risk area in Italy. Geospatial Health.

[bib25] Villeneuve P.J., Burnett R.T., Shi Y., Krewski D., Goldberg M.S., Hertzman C., Chen Y., Brook J. (2003). A time-series study of air pollution, socioeconomic status, and mortality in Vancouver, Canada. J. Expo. Anal. Environ. Epidemiol..

[bib26] Willocks L.J., Bhaskar A., Ramsay C.N., Lee D., Brewster D.H., Fischbacher C.M., Chalmers J., Morris G., Scott E.M. (2012). Cardiovascular disease and air pollution in Scotland: no association or insufficient data and study design?. BMC Public Health.

[bib27] Wong C.M., Ou C.Q., Chan K.P., Chau Y.K., Thach T.Q., Yang L., Chung R.Y.N., Thomas G.N., Peiris J.S.M., Wong T.W., Hedley A.J., Lam T.H. (2008). The effects of air pollution on mortality in socially deprived urban areas in Hong Kong, China. Environ. Health Perspect..

